# In Vitro Investigation of the Effect of Silanizing on the Microshear Repaired Bond Strength of Short-Fiber Composites after Surface Treatment with an Er;Cr:YSGG Laser, Sandblasting, or Bur: Effectiveness of silanizing on the repaired bond strength of Short-fiber composite

**DOI:** 10.31661/gmj.v13iSP1.3646

**Published:** 2024-12-29

**Authors:** Faramarz Zakavi, Nasrin Mohamadi, Azita Kaviani

**Affiliations:** ^1^ Department of Restorative Dentistry, School of Dentistry, Ahvaz Jundishapur University of Medical Sciences, Ahvaz, Iran

**Keywords:** Microshear Bond Strength, Short Fiber-Reinforced Composite, Surface Treatment, Laser Treatment, Silanization

## Abstract

**Background:**

This study aimed to evaluate the effect of various interfacial surface treatments on the repair micro shear bond strength (μSBS) of aged short fiber-reinforced composite (SFRC).

**Materials and Methods:**

The substrate and repair composite material used in the fabrication of eighty samples were SFRC (Ever-X posterior, GC). Based on the mechanical roughening method, the samples were divided into four categories: diamond bur, sandblasting, laser treatment, and a control group. Then, they were split into two groups using the chemical conditioning method: those using a universal bonding that contains silane and those using a separate silane step before the universal adhesive that contains silane. After 40 days of immersion in distilled water at 37°C, the specimens were removed. Universal testing machine was used to implement SBS testing. Using field emission scanning electron microscopy (FESEM), the surface topography of the composite material was examined after the roughening procedures.

**Results:**

Althoughthe study revealed that there was no statistically significant variance in μSBS between groups (P 0.05), but compared between the groups in which silane used, laser has made a significant difference (P 0.05 ). Similarly, no notable distinction was identified in μSBS when utilizing universal adhesive with or without an additional silanizing step (P 0.05),

**Conclusion:**

According the data, surface preparation method dosen't affect the repaired μSBS of SFRC composite. If using silane before universal adhesive, laser preparation is effective on increasing bond strength. Also, adding a separate silanization step before using the silane-containing universal adhesive did not enhance the μSBS and isn't necessary.

## Introduction

With a failure rate of 1% to 4% per year compared to indirect restorations, composite resins are now the most popular restorative material for direct anterior and posterior restorations. This is due to their esthetic properties, adhesion to tooth hard tissues, minimal intervention dentistry strategies, low cost, and adequate clinical performance [1][2][3]. Despite these advantages, physicians had to replace them due to problems such as discoloration, secondary cavities, microleakage, wear, and margin ditching [4]. It was reported that dentists commonly renovate failed restorations 10 years after placement [5]. The reinforcing phase of composites is extensively studied to enhance their viability for high-stress applications. Various approaches are explored like altering filler type, size, and silanization [6][7][8]. Existing research has indicated that the use of short glass fibers is one of the more effective methods for reinforcing composite materials [7][9][10]. In 2019, a specialized short fiber-reinforced composite (SFRC) product called Ever X Posterior (GC Europe) was introduced. The inorganic barium biosilicate fillers and randomly aligned E-glass fibers make up this composite’s matrix. Minimizing polymerization shrinkage and recreating the material’s ability to absorb stress like natural dentin were the primary goals of its development [10][11]. Ever X Posterior’s SFRC formulation is ideal for usage in high-stress areas since it reduces the likelihood of cracks by limiting their formation and propagation [10]. Studies have demonstrated that this composite exhibits improved mechanical properties, including enhanced fracture toughness and fatigue resistance [12]. Consequently, there are two schools of thought on the best course of action when dealing with damaged restorations: repair or replacement. As an example, there is an intrusive procedure that weakens the tooth structure; this method is both time-consuming and expensive [13]. On the other hand, there is a more conservative method that aims to preserve the tooth structure, reduce the likelihood of irreparable pulpal damage, increase the restoration’s lifetime, and not use invasive procedures [14]. Achieving stable and strong adhesion is critical for successful composite restorations [15]. There are several ways that have been suggested in the scientific literature to strengthen the bond between composite materials during repairs [16]. Surface treatment, silanization, and adhesive application are the three usual steps in a composite repair approach [16]. In older composite resin and a fresh layer of composite material can be optimally bonded using this methodical procedure. However, it is important to note that bonding to aged or contaminated composite surfaces can be quite unpredictable. Studies have indicated that in such repair scenarios, the original cohesive strength of the composite may be significantly reduced, by as much as 25% to 80% [17][18].

Nevertheless, a standard procedure for treating aged composite surfaces has yet to be determined [19][20]. Compared to bonding agents, surface roughness in composites, which results in micro- and macro-mechanical retention, is the most important factor in repair strength, according to most research [21][22]. Diamond burs, which is the most common method used Clinically, or air abrasion with aluminum oxide (Al2O3) sandblasting can promote repair bond strength; nevertheless, there is disagreement about the utilization of aluminum oxide for appropriate surface treatment [23][24][25]. Surface preparation with Erbium lasers before repair has recently been presented as an innovative alternative to conventional methods.

One such method for surface treatment in composite repair operations is the use of Er, Cr: YSGG laser technology [26]. The 2.78 μm wavelength of this laser type allows it to be efficiently absorbed by both water and hydroxyapatite crystals [26]. Surface treatment of several restorative materials using Er,Cr:YSGG lasers has been studied by researchers. One potential application of this approach is the restoration of composite resins [26][27]. Nevertheless, extensive data on the efficacy and efficiency of treating composite materials’ surfaces with an Er, Cr:YSGG laser to strengthen their repair bonds is noticeably lacking [28].

Dental restorations are slowly but surely adopting the usage of universal, multipurpose adhesives for chemical surface treatments [29]. Bonding, alloying, and ceramics are just a few of the many uses for these multipurpose adhesives. The elimination of the necessity for a separate silanization phase in the repair operation is made possible by the inclusion of silane agents in some universal adhesives [30]. The chemical adhesion between the resin matrix of the fresh composite and the glass filler particles of the aged composite layer is greatly enhanced by silane coupling agents, which aid in the production of siloxane linkages [31]. On the other hand, reports on whether or not an extra silanizing procedure is required when utilizing universal adhesives that include silane are contradictory [32]. Research by Mendes et al. and others has shown that resin composite repair bond strength can be improved with the addition of an additional silane coating [30].

In this study, we looked at how different chemical and mechanical surface treatments affected the repair bond strength of EverX Posterior, a short-fiber reinforced composite. Among the mechanical treatments that were examined were sandblasting, diamond bur preparation, and the application of the Er, Cr:YSGG laser. A universal adhesive containing silane agents was utilized for the chemical treatments, either with or without an extra step of silanization. We set out to verify two things: first, that the three mechanical surface treatment methods would all produce the same repair bond strength (micro shear bond strength, μSBS); and second, that the silane-containing universal adhesive would produce the same result regardless of whether an extra silanization step was used or not. In order to help clinicians choose the best surface treatment protocols for achieving long-lasting and dependable composite-to-composite repair results, the study evaluated different surface preparation techniques. The goal was to shed light on the most effective and efficient strategies for optimizing the repair bond strength of the short-fiber reinforced composite material.

## Materials and Methods

**Table:** Table1. An overview of the used materials, including the Scotchbond Universal plus adhesive, the Ever-X Posterior composite, and the silane coupling agent. The table lists the manufacturers and the detailed compositions of each material.

**Material**	**Manufacturers**	**Composition**
Scotchbond Universal plus adhesive (SBU)	3M, Germany	A blend of dimethacrylate resins, including 15-25% Bis-GMA and 15-25% HEMA, along with the functional monomer 10-MDP. The resin system is BPA-derivative-free. Additional components included 5-15% silanized silica filler, 1-5% Vitrebond copolymer, 10-15% water, 10-15% ethanol, a silane coupling agent, and the photoinitiator camphorquinone along with a dual-cure accelerator.
Ever-X posterior composite	GC, JAPAN	Bisphenol A-glycidyl methacrylate (Bis-GMA), polymethyl methacrylate (PMMA), and triethylene glycol dimethacrylate (TEGDMA) were the monomers that were used to create the resin matrix. Short e-glass fibers and barium borosilicate glass particles made up the filler phase that reinforced this resin system; they made up 74.2% of the total weight and 53.6% of the volume of the composite.
Silane coupling agent	Ultradent, Germany	The composition included acetic acid, isopropyl alcohol, and 8% methacryloxypropyl-trimethoxysilane. 7.5% ethyl alcohol, 0.2% chlorhexidine, methacrylic acid, and 2-hydroxyethyl methacrylate (2-HEMA) were used to create the bonding agent known as "Peak Universal Bond."

The Ahvaz Jondishapour University of Medical Science provided ethical approval for this study with the number IR.AJUMS.REC.1402.441. For both the substrate and the repair, we used a short-fiber composite resin material, more especially the EverX Posterior product from the Japanese company GC Corporation. We used 3M’s Scotchbond Universal Plus Adhesive, a universal adhesive with a silane agent, to apply the chemical treatments to the surfaces. Also utilized was a distinct silane coupling agent manufactured in the USA by Ultradent. Tabulated in Table-1 are the particular materials together with their contents.

### Specimens’ preparation

Eighty cylindrical specimens, 6 mm in diameter and 4 mm in height were manufactured for this in vitro experiment. Japanese firm GC Corporation’s EverX Posterior bulk-fill, a short-fiber reinforced resin composite material, was used to build the specimens. A glass slide supported the silicone mold, which contained the composite material. A Mylar® strip served to cover the top surface of the mold. There were two 2-millimeter-thick layers of the composite. The next step was to cure each layer using light, utilizing an O-light Woodpecker LED-E curing equipment made by Woodpecker in Beijing, China. Operating the LED unit at a power of 1000 mW/cm² for 40 seconds was done in line with the manufacturer’s instructions. The curing light had a wavelength ranging from 430 to 480 nm and an intensity of 1250 mW/cm², as confirmed by an LED radiometer from SDI in Australia, just before each curing cycle. The specimens were further polymerized for 20 seconds from the base of the glass slab to guarantee full curing of the composite.

All of the cylindrical specimens were immersed in distilled water and incubated at 37°C for 24 hours after the initial manufacturing. The following tests were conducted only on specimens that did not display any macroscopic abnormalities. After that, the specimens were air-dried for 30 seconds after a 30-second rinsing. After that, the samples were set aside in a jar and kept in distilled water at 37°C for 40 days. The distilled water was replaced regularly to ensure that no microorganisms could be present. Following this period of storage, the specimens were randomly assigned to one of four groups, with a total of twenty specimens per group, to facilitate subsequent testing and evaluation.

### Surface Treatments and Repair Procedure

The effects of several surface treatment methods on specimens of short-fiber reinforced composite were investigated using a multi-factorial approach in the study. Different mechanical surface treatments were applied to four groups of twenty specimens each: The specimens in Group Db were roughened using a fine diamond bur with 46 μm grit. The bur was moved in five forward and five backward directions for 10 seconds while being cooled with both air and water. After taking each sample with a fresh bur, we cleaned them with water and let them dry in the air. For Group Sb, an intraoral air abrasion micro blaster unit was used to sandblast specimens with 50-μm Al2O3 particles at a 45-degree angle at 2.2 bar air pressure for 10 seconds. To ensure a consistent surface preparation, the handpiece was moved at a distance of about 5 mm perpendicular to the specimen surface. In the Er,Cr:YSGG laser treatment, specimens from Group L were subjected to the following parameters: 2.78 nm wavelength, 90 mJ energy, 50 Hz pulse frequency, 60 ms pulse duration, and 4.5 W power. The device was manufactured by Waterlase, Biolase Technology, San Clemente, CA, USA. Under conditions of cooling water at a pressure of 55% and air pressure set at 60% (40-60 ml/min), the laser device was operated in a focused contactless mode with a focal diameter of 0.9 mm, while being held 1 mm away from the composite resin. Nobody did anything special for Group C (Control). Divided into two subgroups of ten specimens each, the first subgroup (Db-U, Sb-U, L-U, C-U) was subjected to Scotchbond Universal Plus Adhesive (3M, USA), while the second subgroup (Db-S-U, Sb-S-U, L-S-U, C-S-U) was preceded by a silane coupling agent (Ultradent, USA). With this all-encompassing experimental approach, we were able to systematically examine how different mechanical and chemical surface treatment methods affected the performance of the short-fiber reinforced composite material.

A single operator with proper training performed all of the surface treatment techniques to guarantee uniformity. The specimens were sprayed with water for 10 seconds after each treatment and then dried with an air spray for 5 seconds. Any impurities or leftover material from the surface modification procedures was removed using this method. Once the specimens were cleaned and dried, they were placed in distilled water to await further examination or testing.

### Scanning electron microscope assessment:

To evaluate the topography of the treated surface alterations, four specimens were examined using FESEM, one from each group. After that, each specimen’s surface was quickly dried with ethanol. Then, it was covered with a 13.06 nm thick coating of gold under vacuum for 21 minutes and 41 seconds. The specimen was then studied with a FESEM (TESCAN MIRA 4, Czech Republic) at magnifications of ×100, 500, and 1000. The study utilized SEM to capture high-resolution images of the surface morphology of the bulk-fill composite specimens after the various surface treatment protocols were applied.

### Adhesive and restorative protocols

Chemical conditioning was applied to subgroups Db-U, Sb-U, and L-U after the mechanical surface treatments. Scotchbond Universal Plus Adhesive (3M, USA) was applied to the specimens in these subgroups according to the directions provided by the manufacturer. After the glue was applied to the surface of the composite resin with an applicator brush, a disposable brush was used to rub the surface for 20 seconds. This made sure that the adhesive penetrated the treated surface evenly. Then, for 5 seconds, a gentle airstream was directed onto the surface. After that, the glue stopped moving, which meant that it had been evenly distributed and that some of the solvent had evaporated. In the end, the specimens that had been treated were light-cured for 10 seconds using the Bluephase Style curing unit (Ivoclar Vivadent, Amherst, NY, USA) in its standard power curing mode, with an output intensity of 1200 mW/cm².

Before applying the universal glue, the surfaces of the subgroups Db-S-U, Sb-S-U, and L-S-U were further prepared. The treated composite surfaces were coated with a silane coupling agent, which was supplied by Ultradent, USA, and applied using a disposable brush. According to the manufacturer’s recommendations, the silane was left to react with the surface for one minute. To help any excess or volatile components of the silane agent evaporate, the surfaces were air-dried for 10 seconds after the treatment. Using the identical method as described for the Db-U and Sb-U subgroups, the pretreated surfaces were coated with the universal adhesive (Scotchbond Universal Plus, 3M, USA) after the silane conditioning. Light curing was applied for 10 seconds using the Bluephase Style curing equipment after the adhesive had been brushed over the surfaces and rubbed for 20 seconds.

The specimens were measured using Tygon tubes (Interlab AS, Istanbul, Turkey) that were 2 mm long and had an inner diameter of 1 mm. After that, a composite cannon was used to pack the Ever-X, GC, Japan, bulk-fill composite resin material into the tygon tubes. The next step was to light-cure each specimen for 20 seconds. When the first curing was complete, the tygon tubes were taken out and the specimens went through another 40-second light-curing cycle. The composite material was polymerized thoroughly as a result of this. Next, the specimens were inspected for the existence of any interfacial flaws or voids in the composite using an optical microscope (Olympus SZ 40, SZ-PT, Japan) set at 25X magnification. Specimens were first prepared, and then a repair composite material—identical to the bulk-fill composite—was applied to their surfaces. We added this extra layer of composite to make it look like a repair job. When the specimens were ready to be examined for their μSBS performance, they were placed in distilled water and kept at 37°C until further use.

### Microshear bond strength test

A universal testing device, made in Germany by ZWICK ROELL, was used to assess the μSBS of the test specimens. Each cylindrical composite resin specimen was meticulously tested by attaching a fine metal wire—0.2 mm in diameter—to the base of the apparatus. At room temperature (23 ± 1°C), the specimens were subjected to force in a direction perpendicular to the adhesive contact, with a constant crosshead speed of 0.5 millimeters per minute. Each sample’s loading was kept constant until it failed. To apply a uniform load across the adhesive contact, the load cell and wire loop were precisely positioned to be as straight and aligned as possible. For every specimen, we recorded the highest force value (in Newtons) that the testing apparatus recorded at the time of failure. The ultimate micro-shear bond strength was determined in megapascals (MPa) by dividing this force measurement by the composite cylinder’s cross-sectional area (in square millimeters). One operator used a stereomicroscope (Olympus SZX10, Japan) at 40X magnification to inspect the specimens’ cracked surfaces following the μSBS testing. There were three types of failure modes identified:

a) Adhesive failure - taking place where the original and repaired composite materials meet.

b) Cohesive failure - inside the original composite material or the repaired one.

c) Mixed failure - failure scenarios that combine cohesive and adhesive.

**Figure-1:**
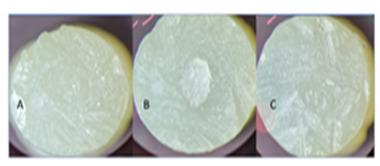
Figure 1. 50X magnification micrographs. (A) Adhesive failure; (B) mixed failure; (C) cohesive failure.

Figure 1(A) depicts an adhesive failure, where the fracture occurred cleanly along the interface between the original and repair composite materials, without any significant cohesive fracture of either substrate. In contrast, Figure 1(B) shows a mixed failure mode, characterized by a combination of adhesive separation at the interface as well as cohesive fracture within one or both of the composite components. Finally, Figure 1(C) illustrates a cohesive failure, where the fracture path propagated entirely through the bulk of either the original or the repair composite, without any visible separation at the adhesive interface.

### Statistical analysis

Statistical analysis was performed on the experimental data using the Macintosh operating system-specific IBM SPSS Statistics software, version 27.0.0.1 (Armonk, NY, USA). An 85% power to detect significant effects was achieved by setting the two-sided alpha error probability at 0.05 in the study. Using separate t-tests, we compared the mechanical surface treatments across the two chemical conditioning groups (one with and one without silane application). Also, to look for variations in the failure mechanisms that were detected, the Mann-Whitney test was used. All of the experimental groups had descriptive statistics computed, such as means and standard deviations. To thoroughly evaluate whether there were any notable variations in the micro-shear bond strength (μSBS) among the various mechanical surface treatment conditions, a one-way analysis of variance (ANOVA) was performed, followed by Tukey’s post-hoc multiple comparisons test. The Kruskal-Wallis test was also used to assess the differences between the separate groups. We used a P-value of less than 0.05 as our significance level for all statistical analyses.

## Results

**Figure-2:**
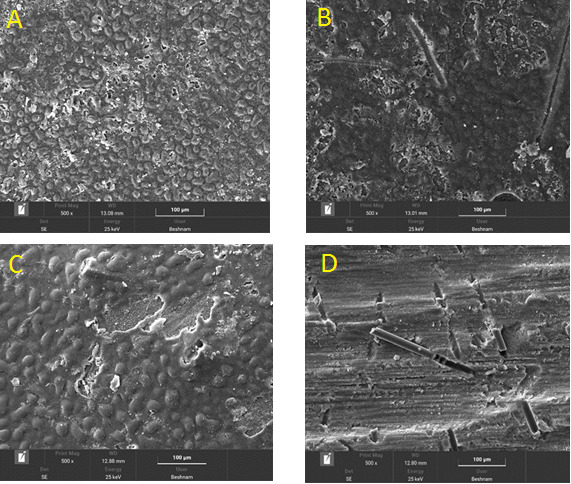
Figure 2. SEM micrograph of composites. (A) control sample; (B) aluminum oxide sandblasting sample; (C) Er,Cr:YSGG laser sample; (D) bur treated sample.

**Table:** Table2. Summary of μTBS Measurements: Mean Values, Standard Deviations, and Statistical Analyses

**Group**	**Subgroup**	**Mean (SD)**	**Tukey`s Multiple Comparisons Test **		**Dunnett 2-sided with Ref. Group **	**Independent Samples t-Test**	
			Subgroup	P-Value	P-Value	Subgroup	P-Value
**Control**	Control+silane+ Bond+Composite	31.30 (9.87)	CBC	0.999	-	-	-
	Control+Bond+Composite	33.07 (10.44)	-	-	-	-	-
**Sandblast**	Sandblast+silane+Bond	28.90 (5.92)	SSB	1.000	0.963	CSBC	0.866
	Sandblast+Bond	30.32 (6.85)	-	-	1.000	CBC	0.855
**Laser**	Laser+Silane+Bond	35.09 (5.60)	LB	0.999	0.750	CSBC	0.615
	Laser+Bond	33.29 (4.17)	-	-	0.988	CBC	1.000
**Bur**	Bur+silane+Bond	25.76 (5.29)	BB	1.000	0.373	CSBC	0.296
	Bur+Bond	24.91 (7.21)	-	-	0.289	CBC	0.134

### μSBS Test and Failure Modes Analysis

Table-2 summarizes the μTBS results for the treatment groups that were assessed. This includes the mean values, standard deviations, and P-values, which stand for statistical significance. Except for the group that had silane treatment, there was no statistically significant difference in micro shear bond strength (p>0.05). There was a significant difference (p<0.05) between the subgroups who had laser mechanical treatment with silane (Laser+Silane+Bond) and the subgroup that received bur+bond, with the former recording the lowest μSBS (24.91), and the latter recording the highest. The μSBS value of the control group was 32.18, which was the same as the laser-treated group. Of all the groups, those receiving bur therapy had the lowest mean μSBS values (25.3833) whereas those receiving laser treatment had considerably higher μSBS values (34.2368). There were no statistically significant variations in the measured μSBS values for the two mechanical treatment groups when using the universal adhesive, with or without the additional silanization phase (p > 0.05), according to the data. Figure-1 displays the distributions of failure modes. Cohesive failure is the worst possible outcome for any group. On the other hand, we have coherent failure modes as follows: C-S-B: 70%, C-B: 77.8%, Sb-S-B: 40%, and Sb-B: 30%. The percentages are as follows: 40% for L-S-B, 55.6% for L-B1, and 60% for B-S-B2. The B-to-B ratio is 62.5%. The reference group also had 70% failures due to cohesiveness within the substrate composite. Adhesive failure is the group’s lowest failure mode.

### SEM Analysis

Compared to the sandblasted samples, the diamond bur-treated group displayed significantly different roughness patterns, as shown in the SEM micrographs. Reflecting the burs’ grinding action, the surfaces that had been abraded by diamond burs showed distinct parallel lines, groove-like patterns, and longitudinal scratches.

On the other hand, the sandblasted surfaces resembled stone in texture and showed a more porous, micropitted, and uneven topography. These sandblasted surfaces were characterized by a high density of microcavities, resulting in a complex and heterogeneous morphological appearance. Interestingly, the subgroup that underwent laser treatment showed a unique surface morphology. These laser-treated surfaces exhibited rounded, crater-like irregularities, devoid of any apparent smear layer. This distinctive surface texture was attributed to the selective material removal and ablation achieved through the laser treatment process.

Figure-2(A) shows the control group, where no surface treatment was performed. This provided a baseline reference for the untreated composite surface. Figure-2(B) depicts the surface of the specimens that underwent aluminum oxide sandblasting. The micrograph reveals a roughened topography with evident pits and irregularities created by the abrasive particles. Figure-2(C) is the surface of the composite samples treated with the Er,Cr:YSGG laser that exhibits a distinct pattern, likely resulting from the thermal and ablative effects of the laser irradiation. Finally, Figure-2(D) showcases the surface morphology of the specimens that were subjected to diamond bur roughening. The surface displays clear grooves and striations created by the mechanical cutting action of the bur.

## Discussion

The results of this study are highly significant for both clinical practice and the advancement of dental materials science. By demonstrating that various surface treatment methods, including laser treatment, sandblasting, and diamond bur, can achieve comparable micro shear bond strength (μSBS) in aged short fiber-reinforced composites (SFRC), the study provides dentists with a range of viable options for restoring dental composites. This flexibility is particularly valuable in clinical settings where resource availability and patient-specific conditions can vary widely. Moreover, the finding that an additional silanization step does not significantly enhance μSBS when using a universal adhesive that already contains silane suggests that simplified and potentially more cost-effective protocols may be sufficient.

Composite dental restorations can be susceptible to eventual failure, regardless of the specific composite material used [33]. However, one of the key advantages of composite resin materials is their capacity for repair and refinishing [17].The reparability of composite restorations is largely dependent on the dynamic changes that occur in the surface chemistry of the cured composite over time. Important considerations include the time it takes for free radical activity to decrease and whether or not there is a surface layer that inhibits oxygen absorption [34]. According to studies, the concentration of free radicals in a polymerized and cured composite peaks within the first twenty-four hours [22]. But this free radical activity fades away during the next two weeks. It is thought that micromechanical interlocking is the main mechanism controlling the bond between an old composite resin and a repair material [22]. On the other hand, scientists and doctors still can’t agree on the best way to treat surfaces to make composite repair techniques work [20].

Whether the old composite and the new repair material can form a strong and long-lasting bond hinges on how well the two materials can mechanically lock together at the interface. For this micromechanical bonding mechanism to work, it is believed that texturizing or roughening the surface of the aged composite is crucial. Despite the abundance of literature on dental composite restoration and repair, there seems to be a dearth of studies focusing on laser-based procedures for fixing short fiber-reinforced composite resins. This study aimed to fill such an information vacuum by testing the impact of several mechanical and chemical surface preparation techniques on the bond strength performance of repaired samples of aged short fiber composite. Bur roughening, air abrasion/sandblasting, and laser ablation with an Er,Cr:YSGG system were among the mechanical surface treatments that were evaluated. In addition, the study investigated the effects of using a universal glue during chemical conditioning, with and without a distinct silanization step.

Although there were no significant variations in micro shear bond strength (μSBS) between the different surface treatment groups (p>0.05) as a result of statistical analysis, there were some noteworthy trends shown by the data. As compared to the other surface preparation approaches, the samples treated with the Er,Cr:YSGG laser showed the highest mean μTBS value of 35.09 MPa, which is quite interesting. The absence of a smear layer on the composite surface and the low power setting of the laser ablation are the reasons the researchers believe to be responsible for this discovery. It is thought that these factors had a good effect on the bonding mechanisms between the repair material and the aged composite [35][36]. This finding agrees with what has been reported before in the literature. Both Murray et al. and Cho et al. found that increasing the laser’s power output improved bonding conditions [37][38]. Furthermore, it was discovered by Etemadi et al. that surface topography may be more successfully created with laser levels below 5 W, allowing for more effective interaction with the composite resin repair material. [39]. The current analysis confirmed the previous findings by showing that the aged composite samples had an appropriately retentive surface when exposed to the Er,Cr:YSGG laser at 4.5 W power. This may have played a role in the better μTBS performance seen in that treatment group.

Depending on the laser’s energy per pulse, the diameter, depth, and volume of the ablated area can be determined by using laser irradiation during surface preparation to ablate the resin composite material. [40]. As the ablation depth increases, there is a risk that the bond between the old substrate and the repair resin composite would be disrupted [36]. On the other hand, a smear layer can be created on the surface of the composite by using surface preparation procedures like sandblasting and diamond bur roughening. Because of this smear layer, the repair’s bond strength can be compromised [41][42]. It’s interesting to note that erbium-based lasers, like the Er,Cr:YSGG system used in this study, have been demonstrated to create surfaces free of smear layer development [35]. This discovery aligns with the current study’s findings, which showed that the laser-treated composite samples did not exhibit any smear layers. Oskoee et al. provided additional evidence for this when they assessed the effectiveness of various laser modalities for composite repair surface treatment. They discovered that the Er,Cr:YSGG laser outperformed Nd:YAG and CO2 lasers in terms of repair bond strength performance, most likely as a result of the lack of a smear layer [43]. It is important to note, nevertheless, that a study by MN Dursun et al. reported conflicting findings, finding that air abrasion/sandblasting produced superior microtensile bond strength outcomes for composite repair than the Er,Cr:YSGG laser and SiC paper groups [44].

When compared to the other surface preparation techniques studied, the laser-based surface treatment strategy showed better repair bond strength performance, according to the current analysis. This result is consistent with the findings of Duran et al., who suggested that laser ablation was a very successful surface treatment method that outperformed even sandblasting techniques in strengthening the bond of composite restoration treatments [36]. Similarly, Murray et al. have suggested that laser surface treatment is a suitable and practical way to enhance the results of bond strength in composite repair situations [37]. Additionally, Rossato et al. found that when used as part of the composite repair process, laser-based surface preparation and bur roughening produced outcomes that were equivalent [18]. When combined with our data, these results from the literature suggest that laser irradiation for surface conditioning is a viable method for maximizing the repair bond strength of old or damaged composite restorations.

Interestingly, the values of μSBS were most similar to those found in the laser-treated group in the control group, which did not undergo any surface preparation. Bond strengths comparable to those attained with laser treatment were seen as a result of the preservation of the oxygen-inhibited layer on the surface of the composite material, which is consistent with the results of many prior research. The bond strength of progressively applied dimethacrylate-based composite materials can be enhanced by adding an oxygen-inhibited layer, as shown in previous studies [45][46][47]. Several studies have concluded that the oxygen-inhibited layer between the successively added dimethacrylate-based composite materials significantly affects the bond strength. This oxygen-inhibited layer seems to act as a glue layer in between the composite layers, improving the chemical interaction between them [45].

Furthermore, short fiber-reinforced composites can increase the thickness or depth of this oxygen-inhibited layer, which in turn strengthens interfacial bonding, according to a study by Bijelic-Danova et al. [45][48][49]. The random orientation of the fibers in SFRC is responsible for its outstanding performance. These fibers control the depth of the oxygen inhibition layer and encourage micro-mechanical interaction between the short protruding fibers on the interlayer surfaces [45][48].

The bond strength can be positively affected by this interlocking process, particularly in stressful situations. In addition, SFRC’s enhanced mechanical properties, particularly its increased fracture toughness, might bolster its resistance to shearing stresses and keep interfacial connections stronger [49].

Laser approaches for repairing short-fiber reinforced composite resin materials seem to be under-discussed in the current literature. [4][17].

Although there is ample documentation regarding the structural and mechanical qualities of these specific resin composites with short fibers, the repair processes for these materials have not been thoroughly studied in dentistry research [50]. The available evidence suggests that further research is necessary to validate and establish effective repair protocols for short-fiber reinforced resin composites. The unique composition and morphology of these materials, with the integration of short reinforcing fibers, may require the exploration of tailored surface preparation and bonding strategies to ensure durable and reliable repair outcomes.

Another key aspect explored in the current study was the role of the adhesive system. There was no discernible variation in the μTBS between specimens that were administered the universal adhesive with or without an additional stage of silanization, according to the results. As a result, the study’s second null hypothesis remained unchanged. These results are consistent with what has been found in other studies [51][52].

It should be mentioned that there are researchers who have offered different perspectives and have suggested that, to increase the bond strength even more, an extra silanization process should be included before using a universal adhesive that contains silane [32]. It is possible that methodological discrepancies are to blame for these contradictory findings, since research that supported the extra silanization step used tensile μTBS testing techniques [53], which can yield distinct results compared to the shear bond strength evaluation employed in this study. It is preferable to decrease the thickness of the adhesive layer while repairing composite restorations, according to relevant studies. Adhesive s with lower viscosity, which can form a thinner adhesive layer, typically provides better bond strength results [54]. A 2024 study revealed that Silanization significantly enhances the repair bond strength of composite resin when used in conjunction with Gluma Bond Universal adhesive and YSGG laser, outperforming other surface treatments and adhesive systems [55].

This study found a significant improvement in bond strength with silanization, which contrasts with our study where silanization did not show a significant improvement. The use of a specific adhesive (Scothbond Universal Plus) and YSGG laser might explain the different results. Another study in 2022 showed that surface silanization slightly enhances the interfacial bonding strength of over-molded hybrid composites of short fiber reinforced polyamide 6 on continuous fiber-reinforced epoxy, but CO2 laser ablation proves more effective [56]. This aligns with our study’s results. However, the effectiveness of CO2 laser ablation suggests that the type of laser used (Er:Cr:YSGG vs. CO2) can significantly impact the results. A similar study in 2021 found that silanized graphene as a nano-inclusion in carbon fiber-reinforced composites significantly enhances mechanical and thermal properties, with 0.5 wt% silanized graphene yielding the best results in terms of tensile strength and modulus of elasticity [57]. According to another study, silanized surface treatments, among other methods, were explored to enhance the shear bond strength between a short fiber-reinforced composite and a particulate-filled composite, with surface roughening by grinding and phosphoric acid etching showing the most significant improvement [58].

This study found that surface roughening methods (grinding and phosphoric acid etching) were more effective than silanization. Our study did not include phosphoric acid etching, which might be a potential area for further investigation.

It is important to note that this study had several significant limitations. Its one-dimensional focus on a particular universal adhesive with silane and a single composite substrate material is a major drawback. Because of this, it’s possible that the results won’t hold water when applied to other composite materials that have different ingredients or different characteristics. In addition, the study was carried out in a controlled laboratory setting, which may not be representative of the real-life clinical setting where restorative materials are utilized. The practicality of the findings is called into question by this disparity. In order to ensure that the results can be effectively applied in practice, further study into the μSBS of repaired composite restorations in real-life clinical situations is required. The findings of this study, which evaluated the effect of various surface treatments on the repair micro shear bond strength (μSBS) of short fiber-reinforced composites (SFRC), have implications for a broader range of dental materials. While the laser treatment method demonstrated the highest μSBS, the lack of significant differences among the groups suggests that the choice of surface treatment may be less critical in achieving adequate bond strength. This insight can be extended to other types of dental composites, such as hybrid or nanofilled composites, which are commonly used in restorative dentistry. Future research could investigate whether the surface treatment methods and silanization strategies used in this study yield similar results with these materials.

## Conclusion

Despite the limitations of the experimental methodology, this in vitro investigation produced some significant discoveries. Laser surface treatment seems to have potential benefits over the other examined approaches when applied before composite repair, among the surface treatment methods that were evaluated. Curiously, the bond strength that was achieved when the oxygen-inhibited layer was kept on the SFRC (without any extra treatment) was similar to what was seen in the laser treatment group.

In addition, there were no notable shifts in the measured μSBS between the laser treatment group and the untreated SFRC group that maintained the oxygen-inhibited layer, regardless of whether the Scotchbond Universal adhesive was applied with or without silane. The results of the SEM analyses confirmed these findings. Further research, including in vivo investigations, is needed to validate these results and evaluate the long-term durability of the repaired composite interfaces under real-world, functional circumstances. Although these conclusions offer valuable insights, it is important to note that the study was designed in vitro, so the findings may not directly apply to clinical scenarios.

## Conflict of Interest

None.

## References

[R1] Demarco FF, Collares K, Correa MB, et al (2017). Should my composite restorations last forever? Why are they
failing?. Braz Oral Res.

[R2] Demarco FF, Collares K, Coelho-de-Souza FH, et al (2015). Anterior composite restorations: A systematic review on long-term
survival and reasons for failure. Dent Mater J.

[R3] Opdam NJ, Van De Sande FH, Bronkhorst E, et al (2014). Longevity of posterior composite restorations: a systematic
review and meta-analysis. J Dent Res.

[R4] Oskoee PA, Oskoee SS, Rikhtegaran S, et al (2017). Effect of various laser surface treatments on repair shear bond
strength of aged silorane-based composite. JLMS.

[R5] Fernández E, Martin J, Vildósola P, et al (2015). Can repair increase the longevity of composite resins? Results of
a 10-year clinical trial. J Dent.

[R6] Xu HH, Quinn JB, Smith DT, et al (2003). Effects of different whiskers on the reinforcement of dental
resin composites. Dent Mater J.

[R7] Garoushi S, Vallittu PK, Lassila LV (2007). Short glass fiber reinforced restorative composite resin with
semi-inter penetrating polymer network matrix. Dent Mater J.

[R8] Zandinejad AA, Atai M, Pahlevan A (2006). The effect of ceramic and porous fillers on the mechanical
properties of experimental dental composites. Dent Mater J.

[R9] Garoushi S, Säilynoja E, Vallittu PK, Lassila L (2013). Physical properties and depth of cure of a new short fiber
reinforced composite. Dent Mater J.

[R10] Garoushi SK, Hatem M, Lassila LV, Vallittu PK (2015). The effect of short fiber composite base on microleakage and
load-bearing capacity of posterior restorations. Acta Biomater Odontol Scand.

[R11] Keulemans F, Garoushi S, Lassila L (2017). Fillings and core build-ups. A clinical guide to fibre reinforced composites (FRCs) in dentistry.

[R12] Molnár J, et al (2022). Fatigue performance of endodontically treated molars restored
with different dentin replacement materials. Dent Mater J.

[R13] Kirsch J, Tchorz J, Hellwig E, et al (2016). Decision criteria for replacement of fillings: a retrospective
study. Clin Exp Dent Res.

[R14] Casagrande L, Laske M, Bronkhorst EM, et al (2017). Repair may increase survival of direct posterior restorations–A
practice based study. J Dent.

[R15] Molnár J, Fráter M, Sáry T, et al (2022). Fatigue performance of endodontically treated molars restored
with different dentin replacement materials. Dent Mater J.

[R16] Altinci P, Mutluay M, Tezvergil-Mutluay A (2018). Repair bond strength of nanohybrid composite resins with a
universal adhesive. Acta Biomater Odontol Scand.

[R17] Ahmadizenouz G, Esmaeili B, Taghvaei A, et al (2016). Effect of different surface treatments on the shear bond strength
of nanofilled composite repairs. J Dent Res Dent Clin Dent Prospects.

[R18] Rossato DM, Bandeca MC, Saade EG, et al (2009). Influence of Er:YAG laser on surface treatment of aged composite
resin to repair restoration. Laser Phys.

[R19] Ritter AV, Sulaiman TA, Altitinchi A, et al (2019). Composite-composite adhesion as a function of adhesive-composite
material and surface treatment. Oper Dent.

[R20] Cuevas-Suarez CE, de Oliveira da Rosa WL, Lund RG, et al (2019). Bonding performance of universal adhesives: an updated systematic
review and meta-analysis. J Adhes Dent.

[R21] Valente LL, Sarkis-Onofre R, Goncalves AP, et al (2016). Repair bond strength of dental composites: systematic review and
meta-analysis. IJAA.

[R22] Loomans BA, Özcan M (2016). Intraoral repair of direct and indirect restorations: procedures
and guidelines. Oper Dent.

[R23] Kiomarsi N, Saburian P, Chiniforush N, et al (2017). Effect of thermocycling and surface treatment on repair bond
strength of composite. J Clin Exp Dent.

[R24] Martos R, Hegedüs V, Szalóki M, et al (2019). A randomised controlled study on the effects of different surface
treatments and adhesive self-etch functional monomers on the immediate
repair bond strength and integrity of the repaired resin composite interface. J Dent.

[R25] Aquino C, et al (2020). Repair bond strength and leakage of non-aged and aged bulk-fill
composite. Oral Health Prev Dent.

[R26] Marx I, Op't Hof J (2002). The Er, Cr:YSGG hydrokinetic laser system for dentistry--clinical
applications. SADJ.

[R27] Soares Machado P, Cadore Rodrigues AC, Trota Chaves E, et al (2022). Surface Treatments and Adhesives Used to Increase the Bond
Strength Between Polyetheretherketone and Resin-based Dental Materials: A
Scoping Review. J Adhes Dent.

[R28] Bayraktar Y, Demirtağ Z, Çelik Ç (2022). Effect of Er:YAG laser pulse duration on repair bond strength of
resin-based and hybrid CAD/CAM restorative materials. JAST.

[R29] Da Rosa WL, Piva E, da Silva AF (2015). Bond strength of universal adhesives: A systematic review and
meta-analysis. J Dent.

[R30] Teixeira Mendes L, Loomans BA, Opdam NJ, et al (2020). Silane Coupling Agents are Beneficial for Resin Composite Repair:
A Systematic Review and Meta-Analysis of In Vitro Studies. J Adhes Dent.

[R31] Matinlinna JP, Lung CY, Tsoi JK (2018). Silane adhesion mechanism in dental applications and surface
treatments: A review. Dent Mater J.

[R32] Silva CL, Scherer MM, Mendes LT, et al (2020). Does use of silane-containing universal adhesive eliminate the
need for silane application in direct composite repair?. Braz Oral Res.

[R33] Ayar MK, Guven ME, Burduroglu HD, Erdemir F (2019). Repair of aged bulk‐fill composite with posterior composite:
Effect of different surface treatments. JERD.

[R34] Atalay C, Yazici AR, Ozgunaltay G (2018). Bond strengths of bulk-fill resin composite repairs: effect of
different surface treatment protocols in vitro. JAST.

[R35] Lizarelli RD, Moriyama LT, Bagnato VS (2003). Ablation of composite resins using Er:YAG laser—comparison with
enamel and dentin. LSM.

[R36] Duran I, Ural Ç, Yilmaz B, Tatar N (2015). Effects of Er:YAG laser pretreatment with different energy levels
on bond strength of repairing composite materials. Photomed Laser Surg.

[R37] Murray AK, Attrill DC, Dickinson MR (2005). The effects of XeCl laser etching of Ni–Cr alloy on bond
strengths to composite resin: a comparison with sandblasting procedures. Dent Mater J.

[R38] Cho SD, Rajitrangson P, Matis BA, Platt JA (2013). Effect of Er, Cr:YSGG laser, air abrasion, and silane application
on repaired shear bond strength of composites. Oper Dent.

[R39] Etemadi A, Shahabi S, Chiniforush N, et al (2015). Scanning electron microscope (SEM) evaluation of composite
surface irradiated by different powers of Er:YAG laser. JLMS.

[R40] Gökçe B, Özpinar B, Dündar M, et al (2007). Bond strengths of all-ceramics: acid vs laser etching. Oper Dent.

[R41] Bektas ÖÖ, Eren D, Siso SH, Akin GE (2012). Effect of thermocycling on the bond strength of composite resin
to bur and laser treated composite resin. LIMS.

[R42] Kimyai S, Mohammadi N, Navimipour EJ, Rikhtegaran S (2010). Comparison of the effect of three mechanical surface treatments
on the repair bond strength of a laboratory composite. Photomed Laser Surg.

[R43] Oskoee PA, Mohammadi N, Chaharom ME, et al (2013). Effect of surface treatment with Er;Cr:YSSG, Nd:YAG, and CO2
lasers on repair shear bond strength of a silorane-based composite resin. J Dent Res Dent Clin Dent Prospects.

[R44] Dursun MN, Ergin E, Ozgunaltay G (2021). The effect of different surface preparation methods and various
aging periods on microtensile bond strength for composite resin repair. Niger J Clin Pract.

[R45] Bijelic‐Donova J, Garoushi S, Lassila LV, Vallittu PK (2015). Oxygen inhibition layer of composite resins: effects of layer
thickness and surface layer treatment on the interlayer bond strength. Eur J Oral Sci.

[R46] AlJehani YA, Baskaradoss JK, Geevarghese A, et al (2016). Shear bond strength between fiber‐reinforced composite and
veneering resin composites with various adhesive resin systems. J Prosthodont.

[R47] Omran TA, Garoushi S, Lassila L, et al (2019). Bonding interface affects the load-bearing capacity of bilayered
composites. Dent Mater J.

[R48] Vallittu PK (1997). Oxygen inhibition of autopolymerization of
polymethylmethacrylate–glass fibre composite. Mater Sci Mater Med.

[R49] Tsujimoto A, Barkmeier WW, Takamizawa T, et al (2016). Relationship between mechanical properties and bond durability of
short fiber‐reinforced resin composite with universal adhesive. Eur J Oral Sci.

[R50] Bijelic-Donova J, Garoushi S, Lassila LV, et al (2016). Mechanical and structural characterization of discontinuous
fiber-reinforced dental resin composite. J Dent.

[R51] Fornazari IA, Wille I, Meda EM, et al (2017). Effect of surface treatment, silane, and universal adhesive on
microshear bond strength of nanofilled composite repairs. Oper Dent.

[R52] Çakir NN, Demirbuga S, Balkaya H, Karadaş M (2018). Bonding performance of universal adhesives on composite repairs,
with or without silane application. JCD.

[R53] Loomans BA, Cardoso MV, Roeters FJ, et al (2011). Is there one optimal repair technique for all composites?. Dent Mater J.

[R54] Eliasson ST, Dahl JE (2017). Effect of curing and silanizing on composite repair bond strength
using an improved micro-tensile test method. Acta Biomater Odontol Scand.

[R55] Dogan E, Cevval Ozkocak BB (2024). The efficacy of Er, Cr:YSGG laser and contemporary universal
adhesive systems on composite resin repair bond strength: an in vitro study. Odontology.

[R56] Ding Y, Tang H, Shi W, et al (2022). Enhancement of interfacial strength of overmolded hybrid
structures of short fiber reinforced polyamide 6 on continuous fiber
reinforced epoxy composites under various surface pretreatments. Polym Compos.

[R57] Shagor RM, Abedin F, Asmatulu R (2021). Mechanical and thermal properties of carbon fiber reinforced
composite with silanized graphene as nano-inclusions. J Compos Mater.

[R58] Lassila L, Tuokko J, Suni A, et al (2022). Effect of interfacial surface treatment on bond strength of
particulate-filled composite to short fiber-reinforced composite. Biomater Investig Dent.

